# Tumor-Associated Platelets Suppress T-cell Function and Promote Immune Evasion in TNBC via the P-selectin/P-selectin Glycoprotein Ligand 1 Pathway

**DOI:** 10.1158/2767-9764.CRC-26-0187

**Published:** 2026-07-10

**Authors:** Margaret R. Smith-Oliver, Deepa Gautam, Grace C. Petrarca, Megan E. Sullivan, Emily M. Clarke, Giovanni Goggi, Milos Spasic, Natalie Kane, Jared Brown, Harvey G. Roweth, Joanna Baginska, Ana C. Garrido-Castro, Patricia Davenport, Sandra S. McAllister, Elisabeth M. Battinelli

**Affiliations:** 1 Division of Hematology, Department of Medicine, Brigham and Women’s Hospital, Boston, Massachusetts.; 2 Harvard Medical School, Boston, Massachusetts.; 3 Division of Newborn Medicine, Boston Children’s Hospital, Harvard Medical School, Boston, Massachusetts.; 4 Department of Data Science, Dana-Farber Cancer Institute, Boston, Massachusetts.; 5 School of Biological Sciences, University of Reading, Reading, United Kingdom.; 6 Dana Farber Cancer Institute, Boston, Massachusetts.; 7 Department of Medical Oncology, Dana-Farber Cancer Institute, Boston, Massachusetts.; 8 Broad Institute of Harvard and MIT, Cambridge, Massachusetts.; 9 Harvard Stem Cell Institute, Cambridge, Massachusetts.; 10 Dana-Farber/Harvard Cancer Center, Boston, Massachusetts.

## Abstract

Tumor-associated platelets (TAP), which are reprogrammed by tumor-derived signals to acquire immunosuppressive properties, represent an emerging mechanism of immune evasion in triple-negative breast cancer (TNBC). Although immune checkpoint inhibitors have shown promise, their efficacy is frequently limited by T-cell exhaustion and therapeutic resistance. In this study, we demonstrate that TAPs, unlike healthy circulating platelets, induce T-cell dysfunction through platelet-derived P-selectin, which engages P-selectin glycoprotein ligand 1 (PSGL-1) on T cells. This interaction promotes immunosuppressive signaling, driving T-cell exhaustion and impairing antitumor cytotoxicity. Using *in vitro* coculture systems and *in vivo* TNBC models, we show that disruption of the P-selectin–PSGL-1 axis, including pharmacologic blockade with the FDA-approved anti–P-selectin antibody crizanlizumab, restores T-cell function and enhances responsiveness to immune checkpoint blockade. Notably, PSGL-1, traditionally recognized for its role in leukocyte trafficking and immune regulation, is co-opted by TAPs to suppress T-cell activity, revealing a mechanism of platelet-mediated immune modulation. These findings establish TAPs as active regulators of antitumor immunity and identify the P-selectin–PSGL-1 axis as a therapeutically actionable target to overcome resistance to immunotherapy in TNBC.

**Significance:**

Tumor-associated platelets help TNBCs evade immune attack by suppressing T-cell function through P-selectin signaling. Blocking this pathway with crizanlizumab restores antitumor immunity and enhances response to immunotherapy, identifying a promising new combination treatment strategy for patients with this aggressive form of breast cancer.

## Introduction

Despite significant advances in cancer research and treatment, breast cancer remains one of the leading causes of cancer-related deaths in women (1). Among its subtypes, triple-negative breast cancer (TNBC) is particularly aggressive, accounting for up to 20% of cases and contributing disproportionately to breast cancer mortality (2, 3). TNBC lacks expression of estrogen receptor, progesterone receptor, and human epidermal growth factor receptor 2 (HER2), rendering it refractory to targeted and endocrine therapies and associated with high rates of metastasis and relapse. Increasing evidence highlights the tumor microenvironment (TME), a complex network of immune cells, stromal components, extracellular matrix, and inflammatory mediators, as a critical regulator of TNBC progression and immune evasion (4–7).

Efforts to enhance antitumor immunity have led to the development of immune checkpoint inhibitors (ICI) targeting programmed cell death protein 1 (PD-1)/programmed cell death ligand 1 (PD-L1) and cytotoxic T lymphocyte–associated protein 4 (CTLA-4), which have transformed cancer therapy. Among breast cancer subtypes, TNBC exhibits relatively higher immunogenicity and is therefore uniquely eligible for first-line immunotherapy (8–11). However, clinical responses remain limited, with many patients exhibiting primary or acquired resistance (12–15). Although multiple mechanisms contribute to ICI failure, emerging evidence implicates platelets as active regulators of immune responses within both the TME and systemic circulation (2, 16). Importantly, platelet-mediated immune modulation may extend beyond the tumor site to influence circulating T-cell function and systemic antitumor immunity.

Traditionally recognized for their role in hemostasis, platelets are increasingly appreciated for their capacity to modulate tumor progression and immune function. Elevated platelet counts correlate with poor prognosis across multiple cancer types (2, 17, 18), and tumor-associated platelets (TAP) have been shown to engage directly with immune cells through adhesion molecules such as P-selectin and its ligand P-selectin glycoprotein ligand 1 (PSGL-1), influencing leukocyte trafficking, T-cell differentiation, and activation (19–21). These findings suggest that platelets are not passive bystanders but active participants in tumor-driven immunosuppression. Importantly, platelet–T-cell interactions are not restricted to the TME but may also occur within the circulation and at metastatic sites, where platelets are well positioned to modulate systemic immune responses.

Upon interaction with tumor cells, platelets undergo phenotypic reprogramming into TAPs, adopting a protumoral and immunosuppressive profile characterized by enhanced secretion of cytokines and increased expression of surface molecules (22–25). TAPs release transforming growth factor β (TGFβ), which promotes epithelial–mesenchymal transition and suppresses effector T-cell function (26, 27). In parallel, platelet-derived PD-L1 has emerged as a contributor to T-cell inhibition in both tumor-localized and circulating compartments (28). We have previously demonstrated that TAPs form aggregates with tumor cells, shielding them from immune detection through upregulation of PD-L1 and phosphorylated epidermal growth factor receptor, thereby promoting immune evasion (24). Together, these features position TAPs at the intersection of tumor survival, metastasis, and immune escape.

P-selectin, a transmembrane glycoprotein expressed by activated platelets and endothelial cells, is rapidly translocated to the cell surface following platelet activation (29, 30). Under physiologic conditions, it mediates leukocyte adhesion through binding to PSGL-1 (31). However, in cancer, P-selectin contributes to tumor progression by facilitating tumor cell–platelet aggregation, protecting tumor cells from immunosurveillance, and promoting metastatic dissemination (32–36). Analysis of The Cancer Genome Atlas further reveals that elevated expression of the P-selectin–encoding gene (*SELP*) is associated with poor survival across multiple malignancies, including breast cancer (37, 38). These observations raise the possibility that the P-selectin–PSGL-1 axis may also play a direct role in suppressing antitumor immunity.

Effector CD4^+^ and CD8^+^ T cells are central mediators of antitumor immunity and key targets of immunotherapy, yet their function is frequently impaired within the TME by immunosuppressive networks involving tumor cells, regulatory T cells (Treg), and stromal elements (39). TAPs may represent an additional layer of immune regulation through PD-L1 expression, TGFβ secretion, and direct cell–cell interactions. Notably, the P-selectin–PSGL-1 axis has been implicated in skewing CD4^+^ T-cell differentiation toward regulatory phenotypes and impairing effector T-cell activation in inflammatory contexts (40–43). Emerging evidence further suggests that PSGL-1 can function as a negative regulator of T-cell activity, contributing to T-cell exhaustion and diminished antitumor responses (44). Collectively, these platelet-mediated mechanisms may represent an underappreciated barrier to effective immunotherapy in TNBC.

We hypothesized that TAPs promote immune evasion in TNBC by suppressing CD4^+^ and CD8^+^ T-cell function through PD-L1 expression and P-selectin/PSGL-1–mediated interactions, thereby driving T-cell exhaustion and resistance to immunotherapy. To test this, we used *in vitro* coculture systems with murine and human T cells and TAPs, *in vivo* syngeneic TNBC models, and comprehensive immunophenotyping to assess T-cell cytotoxicity, exhaustion markers, and checkpoint expression. Given the central role of P-selectin in platelet–immune interactions, we further evaluated whether its blockade could restore T-cell function. To this end, we utilized crizanlizumab, an FDA-approved monoclonal antibody targeting P-selectin, currently used to prevent vaso-occlusive crises in sickle cell disease (45, 46). In addition, we sought to explore how targeting platelet-mediated pathways may complement existing immunotherapies. Together, these approaches enabled us to investigate platelet-mediated immunosuppression and assess the therapeutic potential of targeting the P-selectin axis in TNBC.

## Materials and Methods

### Sex as a biological variable and study approval

Our study exclusively examined female patients and mice, as approximately 99% of breast cancer cases occur in females (47). Mouse experiments were approved under Brigham and Women’s Hospital Institutional Animal Care and Use Committee protocol #2019N000011; human samples were collected with informed consent under Dana-Farber/Harvard Cancer Center Institutional Review Board approval and in accordance with the Declaration of Helsinki.

### Mice

Female C57BL/6 (The Jackson Laboratory #000664, RRID: IMSR_JAX: 000664 and Charles River Laboratories #027, RRID: IMSR_CRL:027) mice (9 weeks) were injected with 2.5 × 10^5^ AT-3 cells or EO771 cells (PBS) into the mammary fat pad. Once tumors reached 100 mm^3^, mice received PBS, anti–PD-1 (150 μg, intraperitoneally, every 4 days, clone RMP1-14, BioXcell, BP0146, RRID: AB_10949053), RB40.34 (30 μg, intraperitoneally, 3 times a week, BD Biosciences, BDB553742, RRID: AB_2254315), or combination therapy. Tumor volumes were measured using calipers [0.5 (length × width^2^)] thrice weekly before treatment and daily after treatment; *n* = 3 to 5/group (48). For T-cell depletion study, anti-CD8β (clone 53-5.8, BioXcell, BE0223, RRID: AB_2687706) antibodies were diluted in sterile PBS at 1 mg/mL and administered at 100 μL per injection on days 6, 7, and 14 following tumor cell injection (49). Platelets for *in vitro* coculture experiments were isolated from female B6;129S2-Selptm1Hyn/J [*SELP*/*SELL* double knockout (KO), The Jackson Laboratory #008432, RRID: IMSR_JAX:008432] mice and compared with platelets from female C57BL/6J wild-type (WT) mice. Although these mice are on a mixed B6;129S2 background, previous studies indicate that platelet activation and adhesion are largely conserved between B6;129S2 and C57BL/6 strains, supporting that observed effects reflect *SELP*/*SELL* deficiency (50).

### Platelet isolation

As previously described, mouse blood was collected from mice through intracardiac puncture using syringes containing 3.2% w/v sodium citrate. Blood was then diluted 1:1 with Tyrode buffer (20 mmol/L 4-(2-Hydroxyethul)piperazine-1-ethane-sulfonic acid (HEPES), 138 mmol/L NaCl, 2.9 mmol/L KCl, 1 mmol/L MgCl_2_, 0.36 mmol/L NaH_2_PO_4_, and 5 mmol/L glucose, pH 7.4). To separate platelet-rich plasma (PRP) from red blood cells (RBC) and white blood cells, diluted blood was centrifuged at 200 × *g* for 10 minutes at room temperature. For platelet isolation, prostaglandin E1 (PGE1; 1 μmol/L; Sigma-Aldrich #P5515) was added to PRP, which was then centrifuged at 400 × *g* for 10 minutes at room temperature. The resulting platelet pellet was resuspended in Tyrode buffer and the platelet concentration normalized to 2 × 10^8^/mL using a Cytek Aurora flow cytometer. The isolated platelets were utilized within 3 hours of blood collection to ensure their functionality (51).

Citrated human whole blood was centrifuged at 177 × *g* for 20 minutes to obtain PRP. PRP was supplemented with PGE1 (1 μmol/L, Sigma-Aldrich #P5515) and centrifuged at 1,000 × *g* for 5 minutes to pellet platelets. The platelet pellet was resuspended in washing buffer (20 mmol/L HEPES, 138 mmol/L NaCl, 2.9 mmol/L KCl, 1 mmol/L MgCl_2_, 0.36 mmol/L NaH_2_PO_4_, and 1 mmol/L ethyleneglycol-bis(B-aminoethyl)-N,N,N′,N′-tetraacetic acid (EGTA), supplemented with 5 mmol/L glucose, pH 6.2) and centrifuged again at 1,000 × *g* for 5 minutes. The platelets were then resuspended in Tyrode buffer (identical to the washing buffer but without EGTA; pH 7.4). Platelet counts were normalized to 2 to 4 × 10^8^/mL using a spectral flow cytometer (Cytek Aurora), and platelets were used within 3 hours of collection to preserve functionality.

### Flow cytometry

Platelets (2 × 10^8^/mL) were stained under three conditions: resting, TAPs, or collagen-related peptide (CRP) activated (Pplus Medical #CRP-A0.5-WIN03, 1 μg/mL). Antibodies included CD62P (RB40.34), CD274 (10F.9G2), galectin-9 (RG9-35), I-Ab (AF6-120.1), CD86 (GL1), and galectin-3 (M3/38). Samples were fixed in 2% paraformaldehyde (Thermo Fisher Scientific #J19943-K2) and analyzed on Cytek with FlowJo v10 (RRID: SCR_008520).

Mouse spleens were processed into single-cell suspensions (RPMI + 10% FBS, 70-μm strainer; Thermo Fisher Scientific), RBC-lysed, and stained with Zombie NIR (BioLegend #423105) plus antibodies (Supplementary Tables S1 and S2; ref. 52). Human peripheral blood mononuclear cell (PBMC; 10 mL blood, Ficoll separation, and RBC lysis) were stained similarly (Supplementary Table S3). Intracellular staining used fixation/permeabilization kits (Thermo Fisher Scientific #00-5523-00 or BioLegend) with markers including IFNγ, granzyme-B, TOX, T-cell factor (TCF) 1, and FOXP3. All data were acquired using Cytek and FlowJo; gating is given in Supplementary Figs. S1 and S2.

### T-cell isolation and activation

Mouse CD3^+^ cells were isolated (EasySep #19851) from AT-3–bearing spleens, cultured in RPMI + 10% FBS, 10 mmol/L HEPES, 100 U/mL penicillin-streptomycin, and activated with IL2 (Thermo Fisher Scientific #212-12-20UG) and Dynabeads CD3/CD28 (Thermo Fisher Scientific #11456D, 1:1) for 48 hours. Human CD3^+^ cells were enriched from PBMCs (EasySep #17951), cultured similarly, and activated with IL2 (PeproTech #200-02) and Dynabeads CD3/CD28 (Thermo Fisher Scientific #11131D). T cells were isolated from AT-3 tumor-bearing mice and used for both *ex vivo* cytotoxicity and suppression assays to ensure experimental consistency.

### T-cell–platelet coculture and cytotoxicity

CD3^+^ T cells were cultured for 48 hours in serum-free RPMI with (i) T cells alone, (ii) tumor cell–conditioned media (TCM), (iii) CRP, (iv) resting platelets, (v) CRP-activated platelets, or (vi) TAPs (T-cell:platelet ratio 1:250 (42). Crizanlizumab (selleckchem #A2374), RB40.34 (BD BDB553742), and anti–PSGL-1 (BioXcell #BE0186) were used as drugs for *in vitro* experiments. For cytotoxicity, T cells were cocultured with AT-3 cells (3,000/well, seeded 24 hours before) at 10:1 for 16 hours. Apoptosis was measured using caspase 3/7 (Thermo Fisher Scientific #C10423), Zombie NIR, and CD3.

### Transwell T-cell and platelet coculture experiments

CD3^+^ T cells and platelets were isolated accordingly and cultured in a Transwell under the conditions specified below in polycarbonate filters (Thermo Fisher Scientific #NC2545699) with all cultures maintained in Gibco serum-free RPMI: (i) T cells with resting platelets (prepared according to platelet isolation protocol) or (ii) T cells with tumor-associated platelets (TAP; prepared according to platelet isolation protocol). Platelets were added to the bottom well and T cells in the upper chamber with a T-cell:platelet ratio of 1:250. All cultures were maintained in Gibco serum-free RPMI at 37°C with 5% CO_2_ for 48 hours. Following incubation, the platelets in the lower chamber and isolated T cells in the upper chamber were collected separately.

### Cell culture and TCM preparation

AT-3 cells were purchased from ATCC (Sigma-Aldrich #SCC178; RRID: CVL_VR89). EO771 (ATCC CRL-3461; RRID:CVCL_GR23) and MDA-MB-231 (ATCC HTB-26; RRID:CVCL_0062) were provided by Dr. Sandra McAllister. All cells were maintained in DMEM + 10% FBS + 1% pen/strep. For TCM, confluent AT-3 or MDA-MB-231 cells were washed, incubated 24 hours in serum-free RPMI, centrifuged (1,000 × *g*, 5 minutes), and supernatants stored at −80°C. Tumor cell lines were species-matched to the platelet and T-cell source (murine AT-3 for mouse studies and human MDA-MB-231 for human studies) to preserve physiologic interactions and avoid cross-species artifacts. Cells between passages 3 and 10 after thawing were used for all experiments and were cultured for no longer than 8 weeks following thawing. All cell lines included in this study were routinely tested and found negative for *Mycoplasma* contamination, with the last test performed on May 16, 2024, using *Mycoplasma* testing by IDEXX BioAnalytics.

### Statistical analysis

All statistical analyses were performed using GraphPad Prism v10 (GraphPad Software, RRID: SCR_002798). Flow cytometry data were expressed as fold change relative to the indicated control (baseline = 1), and statistical significance of these normalized values was assessed using one-sample two-tailed *t* tests or Wilcoxon signed-rank tests, as appropriate. For other comparisons between two matched groups, paired two-tailed Student *t* tests were applied. For experiments involving three or more groups, one-way ANOVA followed by the Tukey multiple-comparison test was used. In cases with predefined pairwise comparisons of interest between independent groups, unpaired two-tailed *t* tests were performed. Data are presented as mean ± standard deviation from three independent biological replicates. Statistical significance is indicated as *, *P* < 0.05; **, *P* < 0.01; ***, *P* < 0.001.

## Results

### Tumor-associated platelets impair effector T-cell function and promote exhaustion

**Figure 1.**
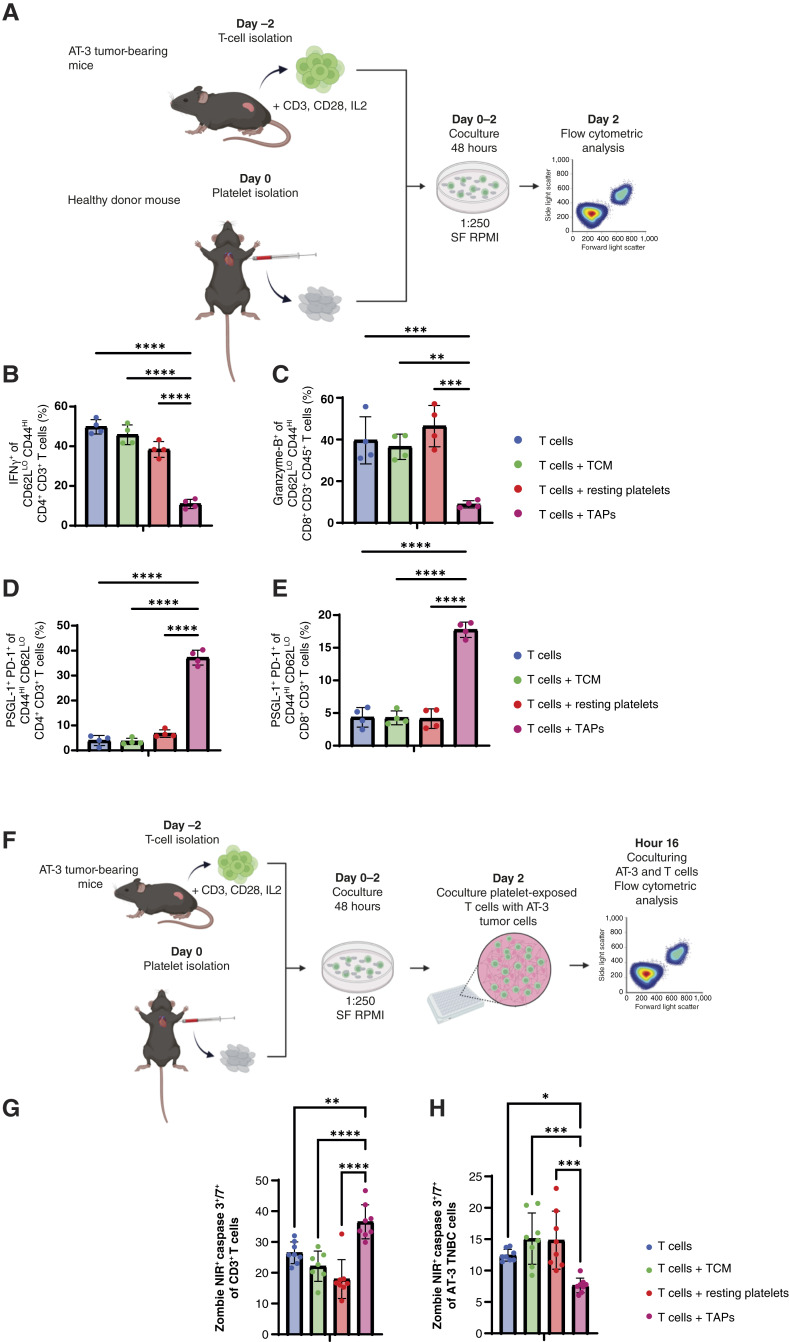
Tumor-associated platelets (TAP) exhaust CD4^+^ and CD8^+^ effector mouse T cells. **A,** Schematic of the T-cell isolation and platelet coculture experiment. Flow cytometry was used to quantify the functionality of (**B**) CD4^+^ and (**C**) CD8^+^ effector T cells and exhaustion of (**D**) CD4^+^ and (**E**) CD8^+^ effector T cells. SF RPMI, serum-free RPMI. **F,** Schematic of the T-cell and AT-3 coculture experiment. Levels of Zombie NIR caspase 3/7 dye detected over 48 hours. Flow cytometry to detect death in (**G**) CD3^+^ T cells and (**H**) AT-3 tumor cells. Data are representative of three independent experiments. Statistical significance was determined using a one-way ANOVA followed by the Tukey multiple-comparison test. *, *P* < 0.05; **, *P* < 0.01; ***, *P* < 0.001; ****, *P* < 0.0001.

To determine whether tumor-associated platelets (TAP) directly contribute to immune evasion, we established a platelet–T-cell coculture system to assess their effect on T-cell function. Platelets were isolated from two healthy mice; one fraction was maintained in serum-free RPMI as resting platelets, whereas the other was incubated for 10 minutes in AT-3 TCM to generate TAPs. As shown previously, platelets become activated in TCM, and we therefore used this system as an *in vitro* model to interrogate TAP–T-cell interactions (25, 53). T cells were isolated from AT-3 tumor-bearing mice, activated with CD3/CD28 and IL2 for 48 hours, and then cocultured under four conditions: (i) T cells alone, (ii) TCM + T cells, (iii) resting platelets + T cells, or (iv) TAPs + T cells (Fig. 1A), enabling assessment of TAP-mediated effects on T-cell activation and cytotoxic function.

Flow cytometric analysis revealed that coculture with TAPs resulted in a significant decrease in functional markers on both CD4^+^ and CD8^+^ effector T cells compared with T cells cocultured with resting platelets or TCM alone (Fig. 1B and C). This suppressive phenotype was not observed in T cells cultured with TCM alone, resting platelets, or CRP-activated platelets, a nontumor platelet activation control, indicating that platelet activation within the tumor context is specifically required to impair T-cell function (Supplementary Fig. S3A and S3B). Furthermore, separation of TAPs and T cells using Transwell assays abrogated the induction of exhaustion-associated phenotypes, demonstrating that direct cellular contact, rather than soluble mediators alone, is necessary for TAP-driven T-cell dysfunction (Supplementary Fig. S3G–S3I). Together, these data establish that TAPs uniquely suppress T-cell function through a contact-dependent mechanism that is not recapitulated by nontumor platelet activation or tumor-derived soluble factors alone.

To identify candidate mediators of platelet-induced T-cell suppression, we profiled platelet surface molecules implicated in immune regulation, including checkpoint ligands (PD-L1 and CD155), adhesion molecules (P-selectin and CD86), galectin-9, and the antigen presentation molecule I-Ab. TAPs exhibited increased expression of PD-L1, P-selectin, galectin-9, and I-Ab relative to resting and CRP-activated platelets (Supplementary Fig. S4A–S4F), suggesting multiple potential inhibitory interactions with T cells (54–59). Given that platelet P-selectin mediates direct adhesion to T cells through PSGL-1, providing a plausible mechanism for the contact-dependent suppression observed, and that PD-L1 functions as a canonical immune checkpoint, we prioritized these pathways based on prior work from our group (17, 24, 36, 60–62). Based on the upregulation of PD-L1 and P-selectin on TAPs, we next assessed whether engagement of these pathways was associated with induction of exhaustion-related phenotypes in T cells.

Consistent with this, T cells cocultured with TAPs displayed significantly increased expression of PD-1 and PSGL-1 on both CD4^+^ and CD8^+^ effector T cells compared with all control conditions (Fig. 1D and E; Supplementary Fig. S3C and S3D). Notably, no increase in exhaustion markers was observed in T cells exposed to platelet-derived soluble factors alone (Supplementary Fig. S3J and S3K), further supporting a contact-dependent mechanism.

To assess functional consequences, T cells previously cocultured under each condition were incubated with AT-3 tumor cells in the presence of a caspase 3/7 reporter (Fig. 1F). T cells exposed to TAPs exhibited increased apoptosis and a marked reduction in tumor cell–killing capacity compared with controls (Fig. 1G and H). In contrast, prior exposure to CRP-activated platelets or platelet-derived soluble factors had no effect on cytotoxicity (Supplementary Fig. S3E, S3F, S3L, and S3M).

Collectively, these data demonstrate that TAPs directly impair T-cell effector function and promote an exhausted phenotype through contact-dependent mechanisms, thereby facilitating tumor-immune escape.

### Human T cells recapitulate platelet-mediated dysfunction

**Figure 2.**
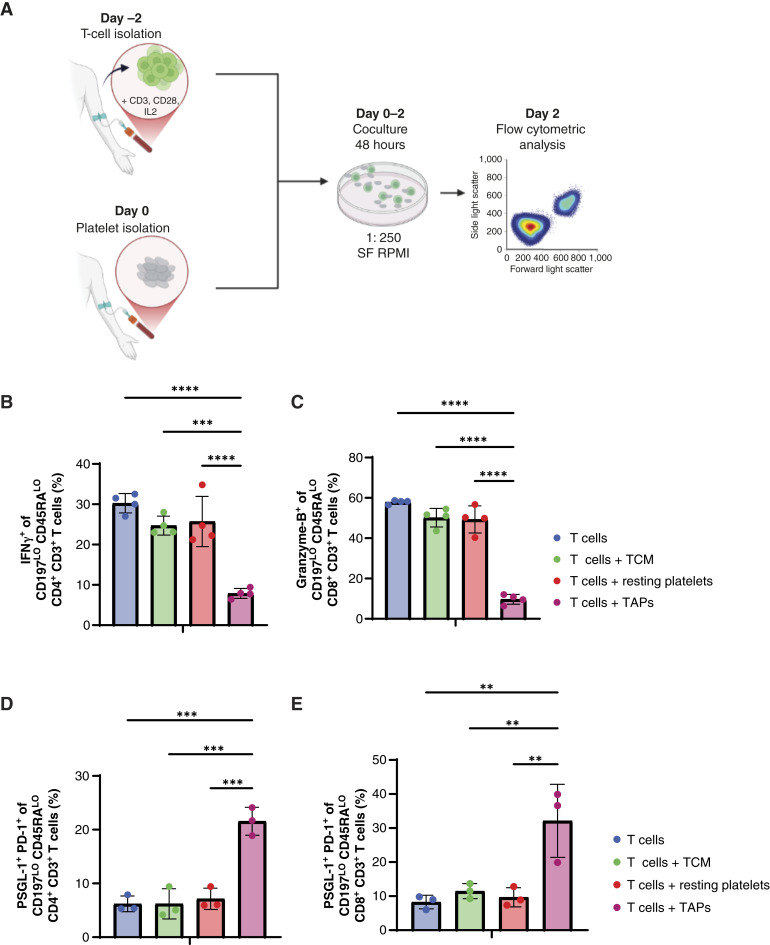
Tumor-associated platelets (TAP) exhaust CD4^+^ and CD8^+^ effector T cells in human. **A,** Schematic of the T-cell isolation and platelet coculture experiment. SF RPMI, serum-free RPMI. Flow cytometry was used to quantify functionality of (**B**) CD4^+^ and (**C**) CD8^+^ effector T cells and exhaustion of (**D**) CD4^+^ and (**E**) CD8^+^ effector T cells in the coculture. Data are representative of three independent experiments. Statistical significance was determined using a one-way ANOVA followed by the Tukey multiple-comparison test. **, *P* < 0.01; ***, *P* < 0.001; ****, *P* < 0.0001.

To evaluate whether the mechanisms of TAP-mediated T-cell suppression are conserved in humans, we conducted coculture experiments using human T cells from healthy donors. Following activation, T cells were cocultured with MDA-MB-231 TCM, resting human platelets, or tumor-activated platelets (TAP; Fig. 2A). Consistent with murine data, TAPs significantly reduced expression of effector molecules in both CD4^+^ and CD8^+^ T cells, whereas resting platelets or TCM alone had no effect (Fig. 2B and C). In parallel, exhaustion markers PD-1 and PSGL-1 were significantly upregulated in T cells cocultured with TAPs (Fig. 2D and E).

Importantly, this phenotype was not recapitulated by CRP-activated platelets, which failed to alter either functional or exhaustion marker expression (Supplementary Fig. S5A–S5D). Moreover, separation of TAPs and T cells using Transwell assays abrogated both functional impairment and induction of exhaustion markers, indicating that direct cell–cell contact is required and that soluble factors alone are insufficient to drive this phenotype (Supplementary Fig. S5E–S5H).

Together, these findings demonstrate that TAP-mediated T-cell dysfunction is tumor specific and contact dependent and that this mechanism is conserved across murine and human systems, underscoring its translational and clinical relevance.

### Loss of P-selectin partially restores T-cell functionality and reduces exhaustion

**Figure 3.**
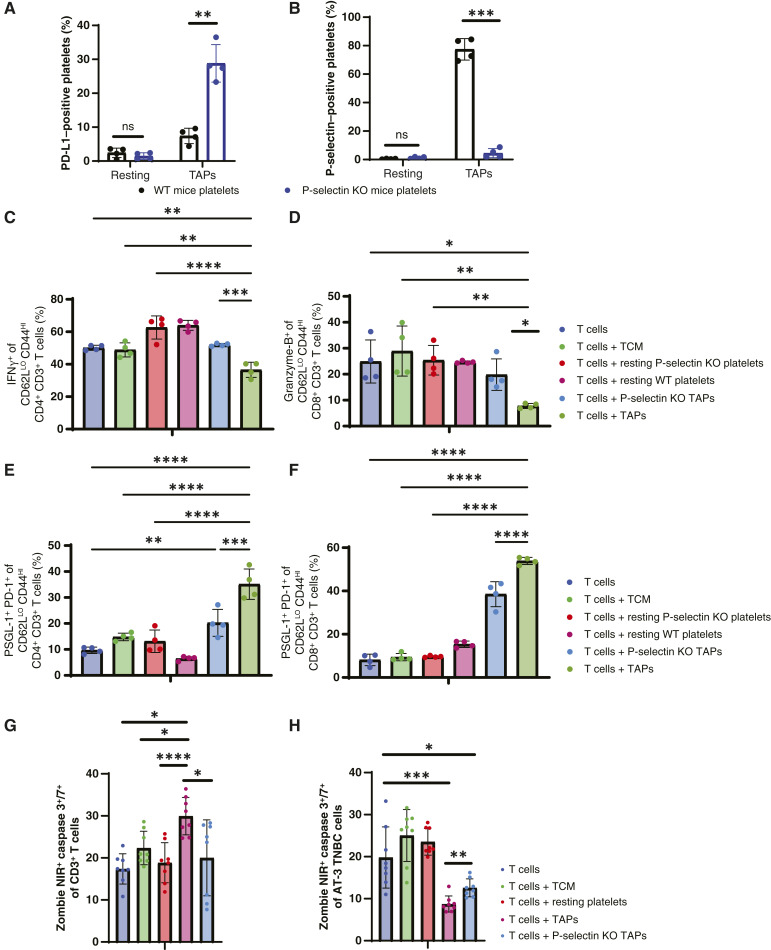
P-selectin KO tumor-associated platelets mitigate T-cell dysfunction. Flow cytometry was used to quantify P-selectin KO TAP levels of (**A**) PD-L1 and (**B**) P-selectin; the functionality of T cells cocultured with P-selectin KO TAPs in both (**C**) CD4^+^ and (**D**) CD8^+^ effector T cells and exhaustion of (**E**) CD4^+^ and (**F**) CD8^+^ effector T cells. Levels of Zombie NIR caspase 3/7 dye detected over 48 hours. Flow cytometry to detect death in (**G**) CD3^+^ T cells and (**H**) AT-3 TNBC cells cocultured with P-selectin KO TAPs. Data are representative of two independent experiments. Statistical significance was determined using a one-way ANOVA followed by the Tukey multiple-comparison test. ns, not statistically significant; *, *P* < 0.05; **, *P* < 0.01; ***, *P* < 0.001; ****, *P* < 0.0001.

Because platelets interact with T cells through the P-selectin–PSGL-1 axis (63), we hypothesized that P-selectin is a key mediator of T-cell dysfunction. Using TAPs from P-selectin KO mice (B6;129S2-*Selp*^*tm1Hyn*^/J), we confirmed that P-selectin KO tumor-associated platelets (P-selectin KO TAP) did not express surface P-selectin yet displayed elevated PD-L1 levels compared with those from WT C57BL/6 mice (Fig. 3A and B). T cells cocultured with P-selectin–deficient TAPs exhibited partial restoration of effector-associated markers in both CD4^+^ and CD8^+^ subsets compared with those cultured with WT TAPs (Fig. 3C and D). Consistent with this, exhaustion markers PD-1 and PSGL-1 were reduced in T cells cocultured with P-selectin KO TAPs (Fig. 3E and F), indicating that the loss of P-selectin mitigates TAP-induced T-cell dysfunction. Importantly, neither CRP-activated platelets nor CRP-activated P-selectin KO platelets altered T-cell functional or exhaustion marker expression (Supplementary Fig. S6A–S6D), demonstrating that platelet activation alone is insufficient to drive these effects and that tumor-conditioned, P-selectin–dependent interactions are required. Functionally, T-cell cytotoxicity assays revealed enhanced AT-3 tumor cell killing and reduced T-cell death when T cells were preconditioned with P-selectin KO TAPs compared with WT TAPs (Fig. 3G and H). Together, these findings identify P-selectin as a central mediator of TAP-induced T-cell suppression while indicating that additional tumor-educated platelet mechanisms contribute to the residual dysfunction, positioning TAPs as multifaceted regulators of T-cell activity within the TME.

### Crizanlizumab blocks the platelet–T-cell interaction and preserves T-cell functionality

**Figure 4.**
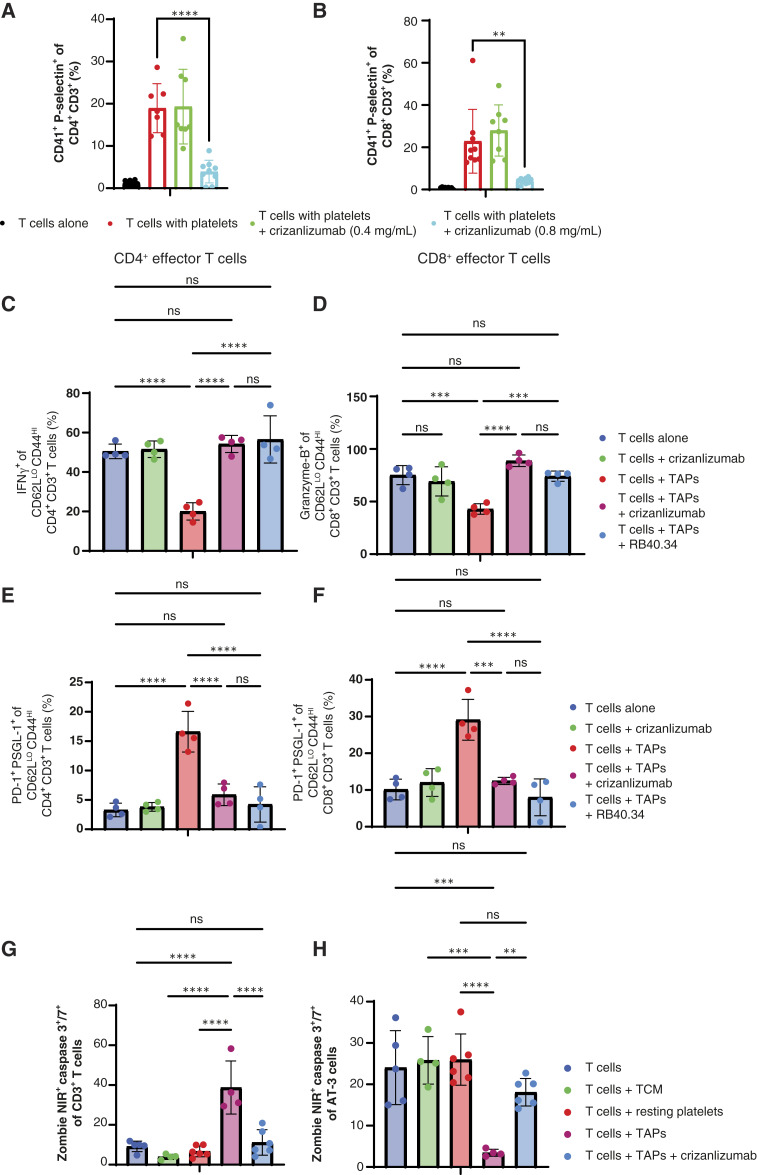
T-cell function preserved with blocked TAP–T-cell interaction through introduction of crizanlizumab. Flow cytometric quantification of platelet–T-cell binding in both (**A**) CD4^+^ and (**B**) CD8^+^ effector T-cell populations. Quantification of the functionality of T cells cocultured with tumor-associated platelets (TAP) and crizanlizumab or RB40.34 in both (**C**) CD4^+^ and CD8^+^ (**D**) effector T cells and exhaustion of (**E**) CD4^+^ and (**F**) CD8^+^ effector T cells. Levels of Zombie NIR caspase 3/7 dye detected over 48 hours. Flow cytometry was used to detect death in (**G**) CD3^+^ T cells and (**H**) AT-3 cells after T cells were previously cocultured with TAPs and crizanlizumab. Data are representative of two independent experiments. Statistical significance was determined using a one-way ANOVA followed by the Tukey multiple-comparison test. ns, not statistically significant; *, *P* < 0.05; **, *P* < 0.01; ***, *P* < 0.001; ****, *P* < 0.0001.

Given the central role of P-selectin in platelet-mediated immune modulation and evidence that P-selectin KO mice exhibit reduced TNBC tumor growth and decreased Treg infiltration, we next tested whether pharmacologic inhibition of this axis could reverse T-cell suppression (64–66). We prioritized targeting P-selectin using crizanlizumab, an FDA-approved anti–P-selectin antibody, as this strategy offers immediate translational relevance and targets the ligand expressed on activated platelets and endothelium, thereby broadly disrupting PSGL-1–dependent interactions across multiple immune cell subsets (46, 67). Crizanlizumab significantly reduced the formation of platelet–T-cell aggregates in both CD4^+^ and CD8^+^ T cells at 0.8 mg/mL, a key interaction through which platelets suppress T-cell effector function (Fig. 4A and B; refs. 68, 69). Using RB40.34, the murine drug used during the preclinical FDA approval process of crizanlizumab, we cocultured T cells with TAPs in the presence or absence of inhibitors (70). Both crizanlizumab and RB40.34 preserved T-cell functionality, as indicated by the maintenance of effector-associated markers (Fig. 4C and D) and prevention of exhaustion marker upregulation (Fig. 4E and F). Furthermore, the inhibition of P-selectin during platelet coculture partially restored tumor cell–killing capacity in cytotoxicity assays (Fig. 4G and H).

To directly test whether these effects are mediated through disruption of the P-selectin–PSGL-1 interaction, we performed coculture experiments in the presence of a blocking anti–PSGL-1 antibody (60, 71). Consistent with a receptor–ligand–dependent mechanism, PSGL-1 blockade recapitulated the protective effects observed with crizanlizumab or RB40.34 (Supplementary Fig. S7A–S7F), demonstrating that interference with either side of the P-selectin–PSGL-1 axis is sufficient to restore T-cell function. Notably, PSGL-1 inhibition was used here to establish mechanistic specificity rather than as a translational strategy, as clinically approved PSGL-1–targeted therapies are not currently available. Collectively, these data establish the P-selectin–PSGL-1 axis as a critical mediator of TAP-induced T-cell suppression and show that its disruption is sufficient to preserve T-cell function, positioning P-selectin blockade with an FDA-approved antibody as a readily translatable therapeutic strategy.

### Crizanlizumab prevents T-cell exhaustion induced by platelets from patients with metastatic TNBC

**Figure 5.**
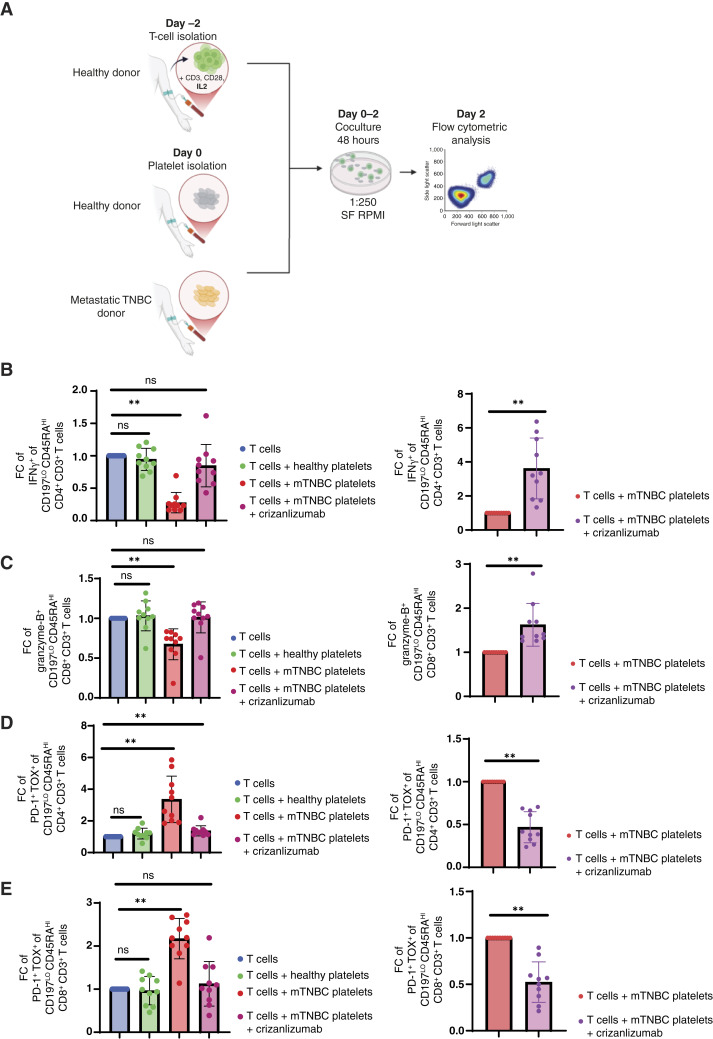
Retained functionality in human CD4^+^ and CD8^+^ effector T cells exhausted by mTNBC platelets through introduction of crizanlizumab. **A,** Schematic of the platelet isolation and T-cell coculture experiment using platelets from healthy donors and patients with mTNBC. SF RPMI, serum-free RPMI. Flow cytometric analysis showing fold change (FC) in (**B**) CD4^+^ T-cell function, (**C**) CD8^+^ T-cell function, (**D**) CD4^+^ T-cell exhaustion, and (**E**) CD8^+^ T-cell exhaustion. For each panel, the FC is calculated relative to T cells cultured alone or relative to T cells cultured with mTNBC platelets, as indicated. Each data point represents one biological sample (*n* = 10 patients). FC values are normalized to the respective control (baseline = 1). Statistical significance was assessed using paired one-sample *t* tests and Wilcoxon signed-rank tests, as appropriate. Error bars represent mean ± SD. ns, not statistically significant; **, *P* < 0.01; ****, *P* < 0.0001.

TAPs have been implicated in immunosuppression, but their effects in human cancer remain poorly defined. To determine whether tumor-educated platelets from patients directly suppress T-cell function and whether this effect can be reversed by P-selectin inhibition, we evaluated platelets isolated from 10 patients with metastatic triple-negative breast cancer (mTNBC) and 10 sex-matched healthy controls. T cells from a healthy donor were activated 2 days before coculture and then cocultured for 48 hours with platelets from sex-matched healthy donors, patients with mTNBC, or mTNBC platelets pretreated with crizanlizumab (Fig. 5A).

Strikingly, platelets from patients with mTNBC alone were sufficient to drive a marked reduction in effector functionality in both CD4^+^ and CD8^+^ T cells compared with healthy donor platelets (Fig. 5B and C). In parallel, mTNBC platelets induced a pronounced exhaustion phenotype, characterized by increased coexpression of PD-1 and TOX in both CD4^+^ and CD8^+^ effector T cells (Fig. 5D and E), demonstrating that patient-derived platelets directly impose T-cell dysfunction and exhaustion independent of other tumor components. Importantly, crizanlizumab treatment during coculture significantly restored effector function in both CD4^+^ and CD8^+^ T cells to levels comparable with those observed with healthy platelets and markedly reduced PD-1^+^ TOX^+^ T-cell frequencies (Fig. 5B–E). These findings show that pharmacologic blockade of P-selectin is sufficient to prevent TAP-induced T-cell dysfunction, even when platelets are derived from patients with advanced disease. Together, these data establish that circulating platelets from patients with mTNBC actively suppress and exhaust T cells and identify the P-selectin–PSGL-1 axis as a clinically actionable pathway to restore T-cell function.

### P-selectin inhibition enhances ICI response in TNBC tumors

**Figure 6.**
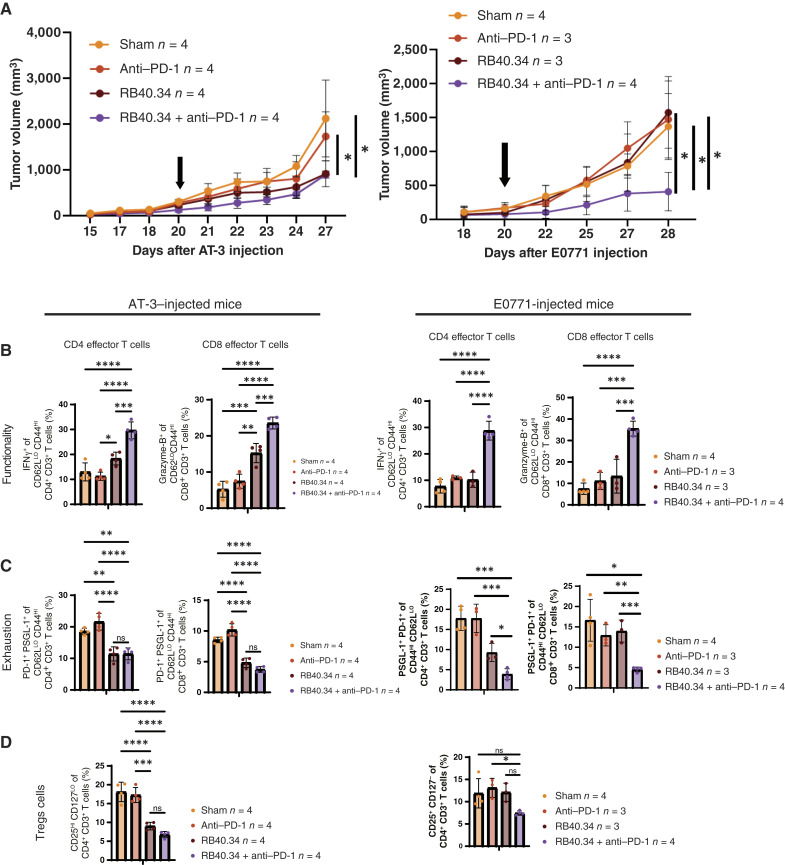
Inhibition of P-selectin increases response to anti–PD-1 in tumor cells. **A,** Tumor volume measurements of mice injected with either AT-3 or EO771 cells that received a combination of RB40.34 and anti–PD-1. Flow cytometry was used to measure functionally active (**B**) CD4^+^ and CD8^+^ effector T cells cocultured with RB40.34 and anti–PD-1, exhaustion of (**C**) CD4^+^ and CD8^+^ effector T cells cocultured with RB40.34, and (**D**) Tregs. Data are representative of two independent experiments. Statistical significance was determined using a one-way ANOVA followed by the Tukey multiple-comparison test. ns, not statistically significant; *, *P* < 0.05; **, *P* < 0.01; ***, *P* < 0.001; ****, *P* < 0.0001.

Consequently, we evaluated RB40.34, the antibody used in the FDA approval process for crizanlizumab (70), for its ability to block P-selectin and reduce tumor growth *in vivo*. We first tested whether the combination of RB40.34 with anti–PD-1 would suppress tumor growth *in vivo*. To ensure that therapeutic responses were not model specific, we tested this combination in two independent syngeneic murine TNBC models with distinct immunologic characteristics (AT-3 and EO771). Mice injected with either AT-3 or EO771 cells that received RB40.34 + anti–PD-1 exhibited significantly reduced tumor growth compared with monotherapy- or sham-treated groups (Fig. 6A). Similar findings were observed in an independent repeat experiment, as provided in Supplementary Fig. S8A and S8B (Supplementary Tables S4 and S5).

Flow cytometric analysis of the spleen revealed that mice treated with RB40.34 + anti–PD-1 had significantly higher numbers of functionally active CD4^+^ and CD8^+^ effector T cells (Fig. 6B). Consistent with this, exhaustion marker expression was reduced in both CD4^+^ and CD8^+^ effector T cells following combination treatment (Fig. 6C). In parallel, we observed alterations in splenic myeloid cell populations, which have been reported to express PSGL-1 (Supplementary Fig. S8C and S8D; ref. 72). Additionally, combination treatment significantly decreased Treg frequencies in the spleen (Fig. 6D). To determine whether T cells are required for therapeutic efficacy, CD8^+^ T cells were depleted beginning 7 days after AT-3 tumor implantation (Supplementary Fig. S8E). Notably, CD8^+^ T-cell depletion completely abrogated the antitumor effects of RB40.34 + anti–PD-1 therapy, demonstrating that tumor control is dependent on CD8^+^ T cells (Supplementary Fig. S8E). Together, these findings demonstrate that combined P-selectin and PD-1 blockade significantly reduces tumor growth across two independent TNBC models by enhancing antitumor immunity. This response is associated with increased CD4^+^ and CD8^+^ effector T-cell function, reduced exhaustion, decreased Tregs, and remodeling of splenic myeloid populations. Importantly, the loss of efficacy following CD8^+^ T-cell depletion establishes that this therapeutic effect is CD8^+^ T-cell dependent, positioning dual P-selectin and PD-1 blockade as a strategy to potentiate T cell–mediated tumor control.

## Discussion

These findings reveal a previously underappreciated role for platelets in shaping systemic antitumor immunity. Specifically, platelets pretreated with TCM from AT-3 or MDA-MB-231 cells induced effector T-cell exhaustion and reduced cytotoxic function, thereby limiting the ability of T cells to kill tumor cells. Importantly, these effects were observed in T cells and platelets isolated from peripheral blood, demonstrating that tumor-activated platelets can induce systemic T-cell dysfunction, with potential consequences for disease progression and therapeutic intervention. By impairing circulating T-cell effector function, tumor-activated platelets may promote systemic immunosuppression and tumor dissemination, suggesting that interventions targeting platelet–T-cell interactions could enhance antitumor immunity and improve patient outcomes (2, 73, 74). This systemic immunosuppression may facilitate metastatic spread, limit disease control, and reduce the efficacy of adoptive T cell–based therapies, all of which depend on intact peripheral immunity.

TCM, enriched in soluble factors secreted by tumor cells, seems to reprogram platelets into immunosuppressive effectors. In this study, we utilized TCM from the AT-3 cell line, an ICI-nonresponsive and highly aggressive model, to better characterize these interactions (75). This reprogramming likely occurs through mechanisms such as the upregulation of surface molecules, including P-selectin, which enhances platelet–leukocyte interactions, and PD-L1, which can promote T-cell exhaustion. The use of CRP-activated platelets as a comparator enabled us to distinguish features specific to tumor-associated platelets (TAP), demonstrating that physiologic platelet activation alone does not impair T-cell function. Rather, it is the tumor-activated state of platelets that drives the observed suppression of T-cell activity. Together, these findings support a mechanism by which TAPs directly interfere with T-cell function, providing new insights into immune evasion in cancer.

In the context of mTNBC, these findings are particularly relevant, as platelets play essential roles in hemostasis, whereas TAPs can become hyperactive, contributing to cancer-associated thrombosis, metastasis, immune evasion, and therapy resistance (24, 76–78). Building on this, our data identify a functional role for platelet-derived P-selectin in promoting T-cell impairment, likely through PSGL-1 engagement, positioning this axis as a potential mediator of systemic immune dysregulation.

Consistent with this model, we observed significantly increased numbers of functional CD4^+^ and CD8^+^ effector T cells in both *in vitro* and *in vivo* settings following P-selectin blockade. *In vitro*, crizanlizumab preserved T-cell function and cytotoxicity. *In vivo*, monotherapy with RB40.34 produced effects comparable with anti–PD-1 treatment, suggesting that platelet-mediated T-cell dysfunction may also occur through alternative pathways, including PD-1/PD-L1 signaling. Notably, crizanlizumab binds the N-terminal domain of P-selectin, whereas RB40.34 targets the lectin domain, suggesting that engagement of distinct functional regions of P-selectin may differentially influence platelet-mediated immune regulation and account for the differences observed between these agents (79, 80). Blockade of either P-selectin or PD-1 reduced tumor growth compared with controls, whereas combined P-selectin and PD-1 inhibition produced the greatest therapeutic response in an ICI-nonresponsive TNBC model. This enhanced response was associated with a significant expansion of pre-exhausted T cells, a subset reported to possess stem-like properties and preferential responsiveness to immune checkpoint blockade (81–85). In addition, combination therapy increased effector T-cell functionality and reduced exhaustion marker expression, supporting a model in which dual P-selectin and PD-1 blockade cooperatively preserves T-cell function.

These results indicate that CD8^+^ T cells are a critical mediator of the antitumor effects observed following P-selectin blockade, positioning CD8^+^ T-cell function as a central downstream effector of platelet–T-cell interactions in this model.

Importantly, these findings are supported by patient-derived data demonstrating that platelets isolated from patients with mTNBC can directly induce T-cell exhaustion, even in the absence of ongoing tumor cell contact. Using healthy donor T cells, we show that TAPs retain a programmed capacity to drive this dysfunctional phenotype. Consistent with this, platelets from patients with mTNBC exhibit increased expression of immunomodulatory molecules, including P-selectin and PD-L1, which likely contribute to their suppressive effects (77, 86). Beyond mechanistic insights, these findings also highlight the potential of platelet-derived markers as accessible, blood-based biomarkers of immunotherapy response. Given the limitations of current biomarkers, such as PD-L1 expression and tumor-infiltrating lymphocyte density (87–90), platelet-associated signatures, including PD-L1 and P-selectin expression, may provide a complementary approach for patient stratification and therapeutic monitoring (77, 91–93).

Although antiplatelet therapies such as aspirin have shown promise, their inconsistent efficacy underscores the need for more precise strategies that selectively disrupt platelet–tumor interactions without impairing normal platelet function. Platelet-derived factors, including cytokines and growth factors, can influence T-cell activation and reshape systemic immune responses (94). Although our Transwell experiments, which physically separated platelets from T cells, enabled the assessment of soluble factors in isolation, we cannot exclude the possibility that the Transwell system itself altered platelet behavior and induced the release of these factors. Further work is needed to fully delineate the contributions of soluble versus contact-dependent mechanisms in platelet-mediated immune regulation. Although we did not directly assess platelet–neutrophil interactions in this study, our findings do not exclude contributions from other P-selectin–PSGL-1–mediated interactions, and future work will be required to delineate the relative roles of different myeloid and lymphoid cell populations in this axis.

Collectively, these data establish TAPs as active regulators of systemic immunity rather than passive bystanders. Moreover, because ICIs targeting PD-L1 may also influence platelet phenotype and function, platelet–T-cell interactions represent an underappreciated determinant of therapeutic efficacy. Targeting platelet-driven immunosuppression may therefore provide a complementary strategy to restore robust T-cell responses while preserving normal hemostatic function in patients with cancer.

Despite these insights, several limitations warrant consideration. Platelets from *SELP*/*SELL* double-KO mice were derived from a mixed B6;129S2 background, whereas WT platelets were obtained from C57BL/6J mice. Additionally, the KO strain used (B6;129S2-Selptm1Hyn/J) lacks both P-selectin (*SELP*) and L-selectin (*SELL*), rather than *SELP* alone; therefore, we cannot fully exclude a potential contribution of *SELL* deficiency to the observed phenotypes. However, L-selectin is predominantly expressed on leukocytes, where it mediates lymphocyte homing and leukocyte rolling on the endothelium, whereas platelets do not express functional L-selectin (95, 96). In contrast, P-selectin is stored in platelet α-granules and rapidly translocated to the surface upon activation, where it stabilizes platelet aggregates and mediates platelet–leukocyte interactions (34, 95). Because our *in vitro* experiments used only isolated platelets without tumor implantation or other *in vivo* manipulations, it is unlikely that *SELL* deficiency substantially influenced these findings, and the observed platelet phenotypes are most plausibly attributable to the loss of *SELP* rather than *SELL*.

Additional limitations apply to the interpretation of our *in vivo* therapeutic studies. P-selectin is expressed not only on platelets but also on activated endothelial cells; therefore, systemic administration of anti–P-selectin antibodies may influence additional processes such as leukocyte trafficking and vascular adhesion. Although our *in vitro* studies using isolated platelets support a direct role for platelet P-selectin in suppressing T-cell function, they do not definitively establish platelet-restricted mechanisms *in vivo*. Nevertheless, the requirement for CD8^+^ T cells *in vivo*, together with our platelet-specific *in vitro* findings, supports a model in which platelet-associated P-selectin contributes substantially to the observed immune modulation, although endothelial contributions cannot be excluded. Furthermore, our immune profiling following therapy was limited to splenic lymphocyte populations and did not directly assess immune or platelet infiltration within the TME. In addition, the limited availability of patient-derived platelets restricted our ability to perform comprehensive functional characterization studies, including platelet activation, aggregation, secretion, and receptor activation assays in samples from patients with TNBC. Future studies incorporating platelet-specific depletion or conditional *Selp* deletion cre models, together with comprehensive analysis of tumor-infiltrating immune and myeloid populations, will be necessary. Although prior studies have examined the role of platelets in tumor progression and immune modulation, including the use of platelet depletion approaches such as anti-GPIbα targeting and the effects of these approaches on metastasis, these approaches have not fully integrated platelet-specific genetic models with comprehensive profiling of tumor-infiltrating immune and myeloid populations (66, 97, 98). Following reanalysis using isotype and background controls, CD155 expression was found to be indistinguishable from background levels and was therefore excluded from downstream analyses, improving the specificity of the platelet phenotyping approach. Because P-selectin–PSGL-1 interactions are also known to regulate platelet–neutrophil adhesion, future studies will be needed to determine the relative contributions of platelet interactions with neutrophils versus T cells in shaping antitumor immunity.

Our *in vitro* TCM models also do not fully recapitulate the complexity of the TME, where platelet activation is shaped by multiple systemic and local factors. Nevertheless, these experiments demonstrate that tumor-derived signals alone are sufficient to activate platelets and suppress effector T-cell function in the circulation. Consistent with this, *in vivo* blockade of P-selectin in combination with anti–PD-1 significantly reduced tumor burden in a preclinical TNBC model, suggesting improved systemic T-cell function rather than directly demonstrating intratumoral immune remodeling. Although these findings indicate that P-selectin inhibition can enhance antitumor immune responses in TNBC, its efficacy in other cancer types remains unknown. Additional studies are needed to determine whether targeting TAP–T-cell interactions represent a broadly applicable immunotherapeutic strategy.

Further investigation is needed to define how platelets and T cells interact within the human TME, including the contributions of cytokines, integrins, and growth factors. Elucidating these pathways may enable strategies that selectively disrupt platelet-driven immunosuppression while preserving hemostatic balance. Together, these findings position platelets as active systemic regulators of antitumor immunity in TNBC and identify P-selectin–mediated platelet–T-cell interactions as actionable targets to enhance immunotherapy efficacy.

## Supplementary Material

Supplementary Figure S1 Gating strategies used to identify different T cell types in mouse

Supplementary Figure S2 Gating strategies used to identify different T cell types in human

Supplementary Figure S3 CRP-activated platelets and platelet-derived soluble factors do not alter murine effector T cell function, exhaustion and viability

Supplementary Figure S4 Platelet expression of immune regulatory molecules

Supplementary Figure S5 CRP-activated platelets and platelet derived soluble factors do not alter human effector T cell function or exhaustion

Supplementary Figure S6 Mouse CRP-activated KO-P-selectin activated platelets do not affect CD4+ and CD8+ Effector T cells

Supplementary Figure S7 PSGL-1 blockade produces similar protective effects against exhaustion in CD4+ and CD8+ effector T cells as P-selectin inhibition

Supplementary Figure S8 Combination of RB40.34 + anti-PD-1 results in response to treatment in TNBC.

Supplementary Table S1 Mouse T-cell exhaustion flow panel

Supplementary Table S2 Mouse broad immune flow panel

Supplementary Table S3 Human T-cell exhaustion flow panel

Supplementary Table S4 AT-3 injected mouse tumor data

Supplementary Table S5 EO771 injected mouse tumor data

## Data Availability

Data are available on request to the corresponding author, E.M. Battinelli (embattinelli@bwh.harvard.edu).
