# Supplementary figures and images for: Tumor-Associated Platelets Suppress T-cell Function and Promote Immune Evasion in TNBC via the P-selectin/P-selectin Glycoprotein Ligand 1 Pathway
Source: Cancer Res Commun. 2026 Jul 10;6(7):1640–55. doi: 10.1158/2767-9764.CRC-26-0187 (PMC13352369; doi:10.1158/2767-9764.CRC-26-0187)

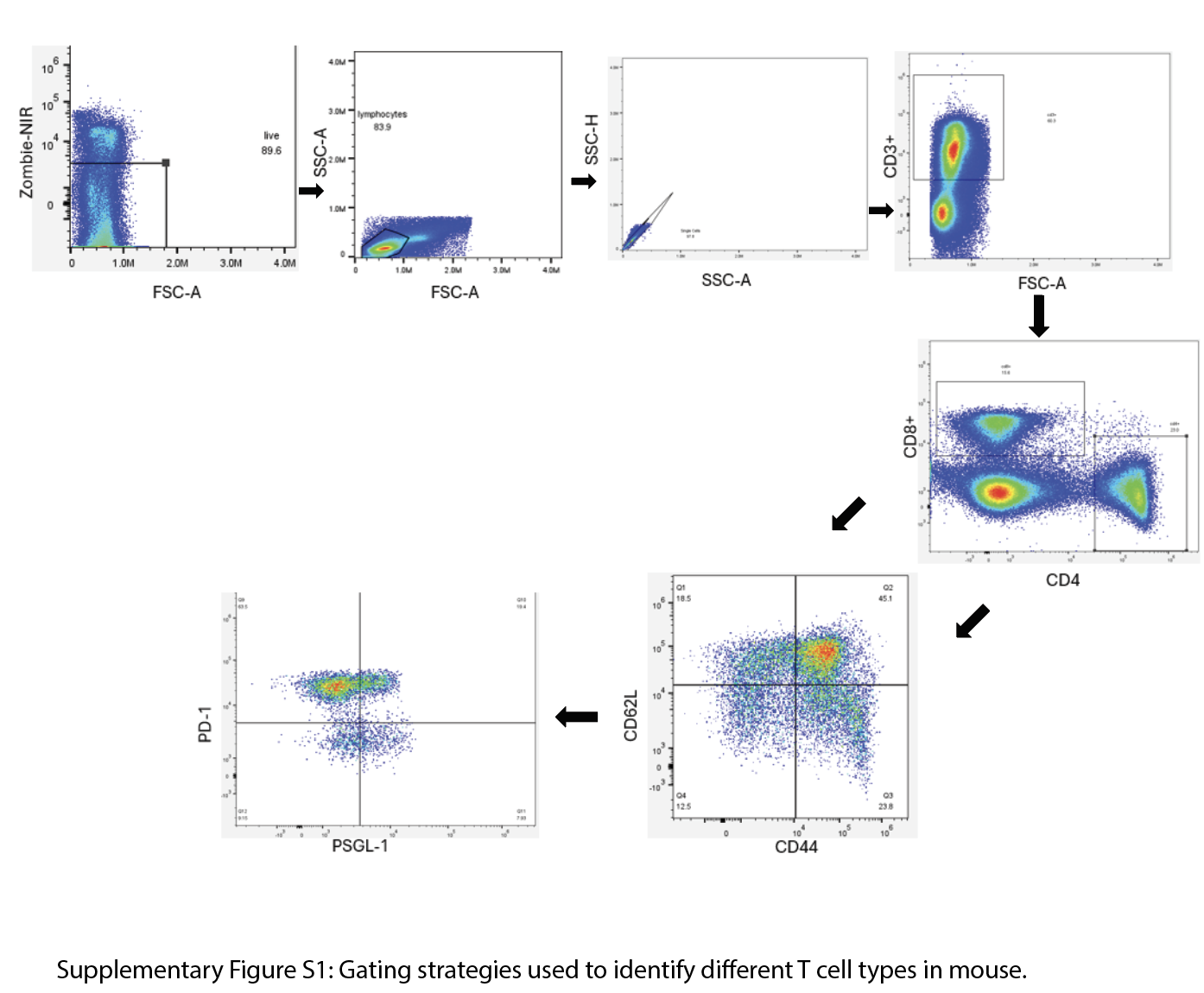

Supplement: Supplementary Figure S1 — Gating strategies used to identify different T cell types in mouse [file crc-26-0187_supplementary_figure_s1_suppsf1.png]

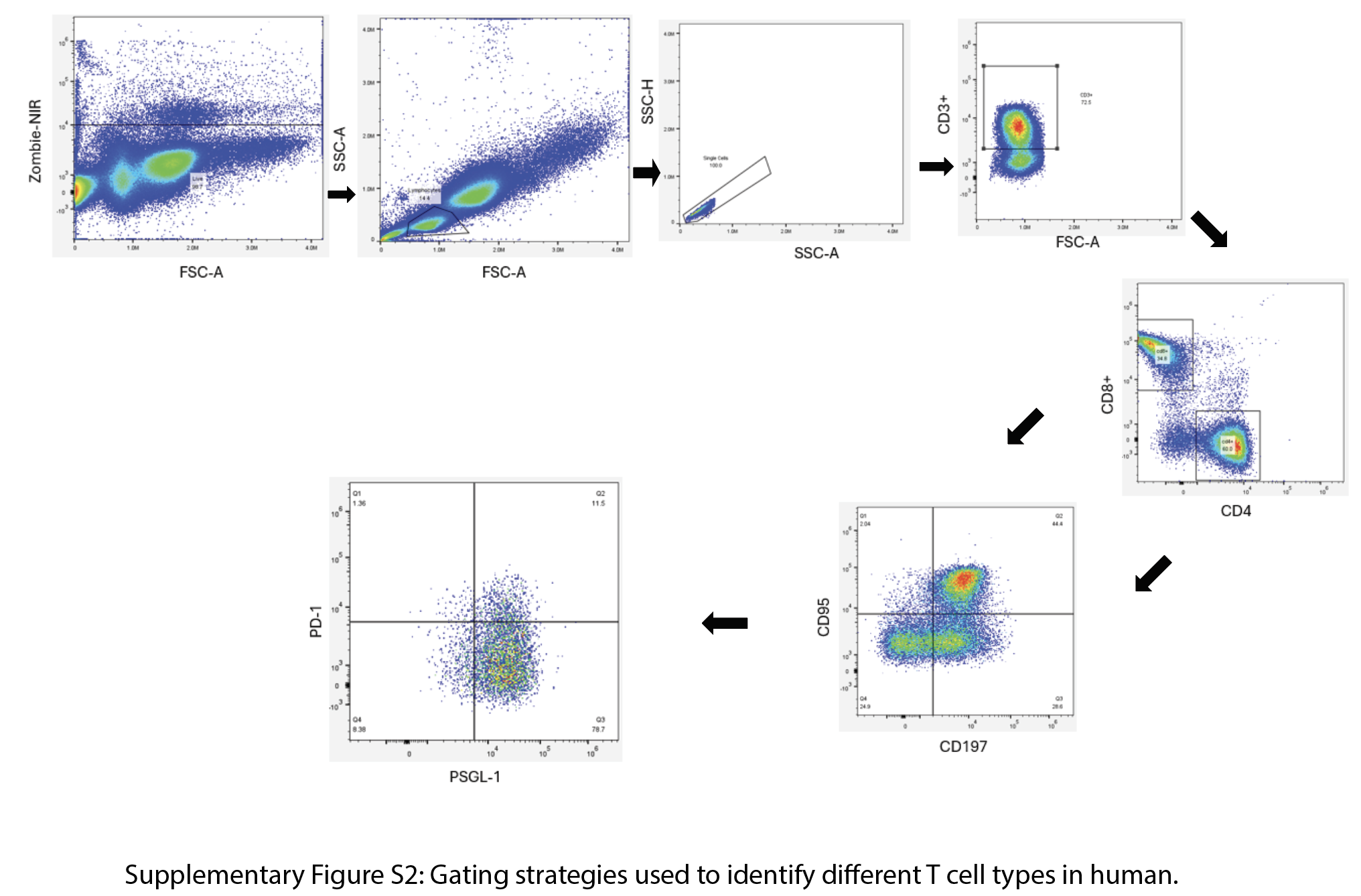

Supplement: Supplementary Figure S2 — Gating strategies used to identify different T cell types in human [file crc-26-0187_supplementary_figure_s2_suppsf2.png]

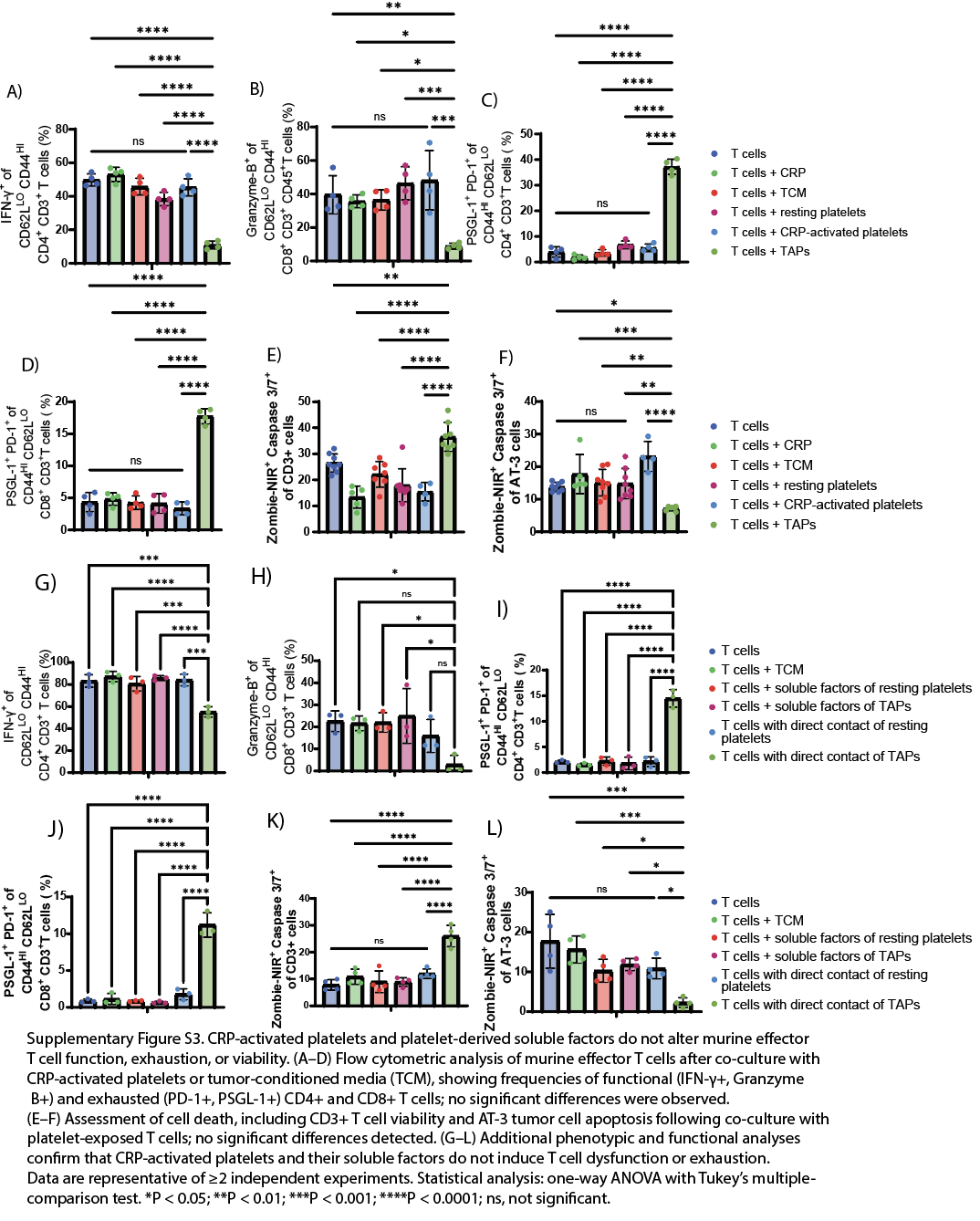

Supplement: Supplementary Figure S3 — CRP-activated platelets and platelet-derived soluble factors do not alter murine effector T cell function, exhaustion and viability [file crc-26-0187_supplementary_figure_s3_suppsf3.png]

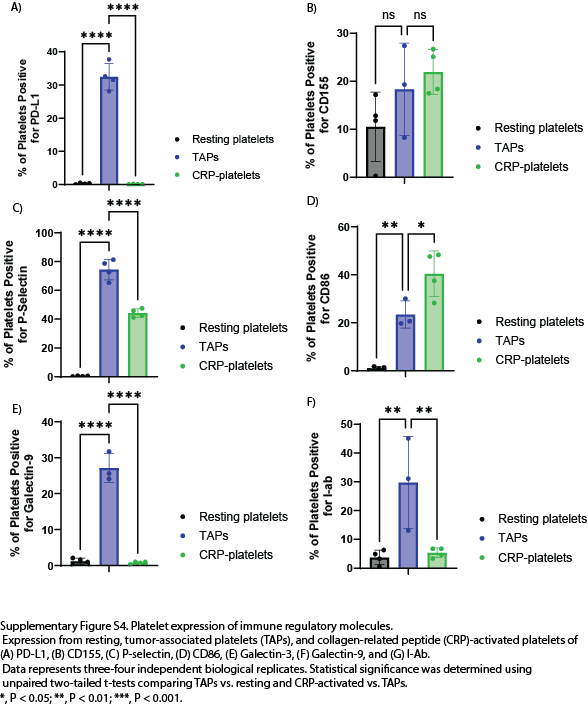

Supplement: Supplementary Figure S4 — Platelet expression of immune regulatory molecules [file crc-26-0187_supplementary_figure_s4_suppsf4.png]

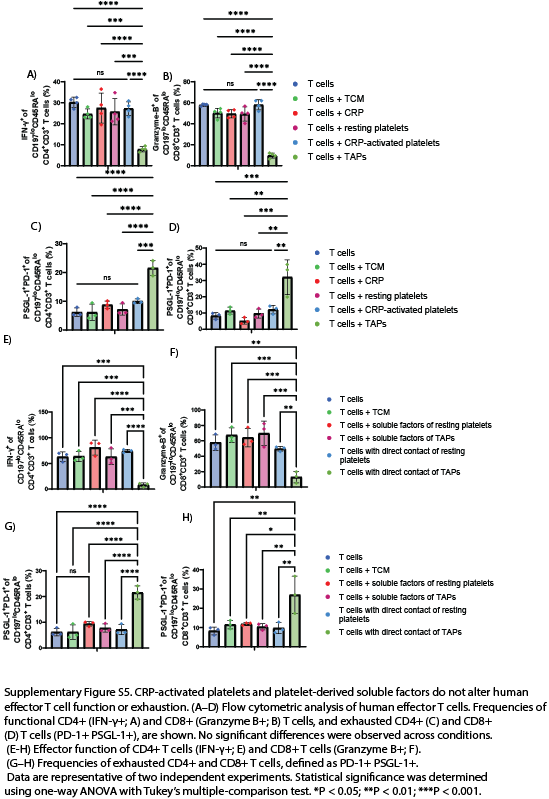

Supplement: Supplementary Figure S5 — CRP-activated platelets and platelet derived soluble factors do not alter human effector T cell function or exhaustion [file crc-26-0187_supplementary_figure_s5_suppsf5.png]

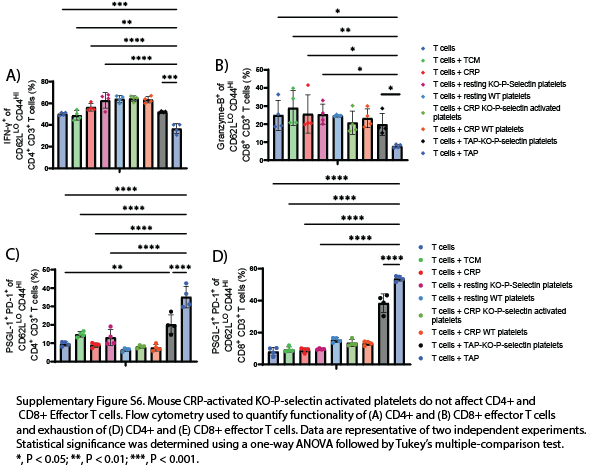

Supplement: Supplementary Figure S6 — Mouse CRP-activated KO-P-selectin activated platelets do not affect CD4+ and CD8+ Effector T cells [file crc-26-0187_supplementary_figure_s6_suppsf6.png]

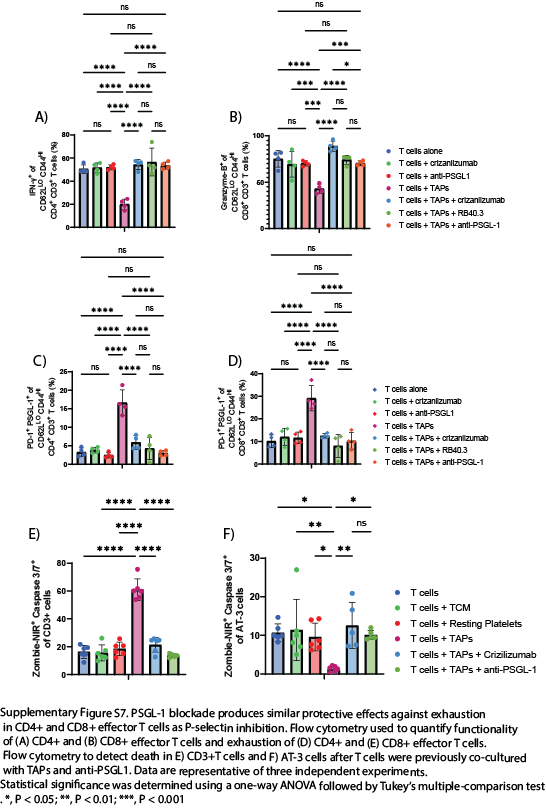

Supplement: Supplementary Figure S7 — PSGL-1 blockade produces similar protective effects against exhaustion in CD4+ and CD8+ effector T cells as P-selectin inhibition [file crc-26-0187_supplementary_figure_s7_suppsf7.png]

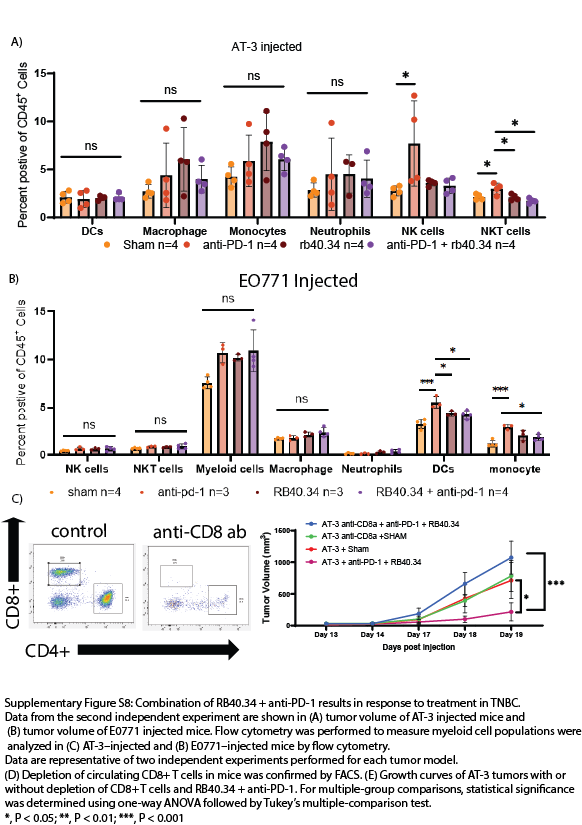

Supplement: Supplementary Figure S8 — Combination of RB40.34 + anti-PD-1 results in response to treatment in TNBC. [file crc-26-0187_supplementary_figure_s8_suppsf8.png]
