# Supplementary material for: Tumor-Associated Platelets Suppress T-cell Function and Promote Immune Evasion in TNBC via the P-selectin/P-selectin Glycoprotein Ligand 1 Pathway
Source: Cancer Res Commun. 2026 Jul 10;6(7):1640–55. doi: 10.1158/2767-9764.CRC-26-0187 (PMC13352369; doi:10.1158/2767-9764.CRC-26-0187)
Supplement: Supplementary Table S3 — Human T-cell exhaustion flow panel [file crc-26-0187_supplementary_table_s3_suppst3.docx]

| Name | Conjugate | Brand | Catalog Number | Clone | Dilution Factor |
| --- | --- | --- | --- | --- | --- |
| CD3 | Alexa Fluor 532 | UCHT1 | ThermoFisher | 58-0038-42 | 1/200 |
| GranzymeB | eFluor450 | N4TL33 | ThermoFisher | 48-8896-42 | 1/200 |
| CD223/LAG-3 | PE/Fire™ 810 | T47-530 | BD | 565716 | 1/200 |
| CD4 | APC-R700 | RPA-T4 | BD | 564975 | 1/200 |
| CD8 | APC-H7 | SK1 | BD | 560179 | 1/200 |
| PSGL-1 | Super Bright 702 | 3g8 | ThermoFisher | CL488-65090100T | 1/100 |
| CD197) | PE-Cy5.5 | 3D12 | ThermoFisher | 35-1979-42 | 1/100 |
| CD25 (T-reg) | Alexa Fluor 488 | M-A251 | BioLegend | 356132 | 1/100 |
| CD279 (PD-1) | PE-Cy7 | EH12.1 | BD | 561272 | 1/100 |
| CD366 (TIM3) | BV786 | 7D3 | BD | 742857 | 1/100 |
| CD45RA | BV650 | HI100 | BD | 563963 | 1/100 |
| CD95 (FAS) | BV480 | DX2 | BD | 746675 | 1/100 |
| Foxp3 | Spark NIR 685 | 206D | BioLegend | 320130 | 1/100 |
| IFN-g | eFluor506 | 4S.B3 | ThermoFisher | 69-7319-42 | 1/100 |
| TCF1 | PE | 7F11A10 | BioLegend | 655208 | 1/100 |
| TIGIT (IC) | BV421 | A15153G | Biolegend | 372709 | 1/100 |
| CD152 (CTLA-4) | PE-eFluor 610 | 14D3 | ThermoFisher | 61-1529-42 | 1/100 |
| TOX | APC | REA473 | Miltenyi Biotec | 130-118-335 | 1/25 |
| Viability | Zombie-NIR |  |  |  | 0 |

**Supplementary Table S3.** Human T-cell exhaustion flow panel
